# Melatonin-Mediated Cytoprotection against Hyperglycemic Injury in Müller Cells

**DOI:** 10.1371/journal.pone.0050661

**Published:** 2012-12-04

**Authors:** Tingting Jiang, Qing Chang, Zhenyang Zhao, Saimei Yan, Ling Wang, Jiyang Cai, Gezhi Xu

**Affiliations:** 1 The Department of Ophthalmology, Eye and ENT Hospital, Fudan University, Shanghai, China; 2 The Vanderbilt Eye Institute, Vanderbilt University Medical Center, Nashville, Tennessee, United States of America; Institut de la Vision, France

## Abstract

Oxidative stress is a contributing factor to the development and progression of diabetic retinopathy, a leading cause of blindness in people at working age worldwide. Recent studies showed that Müller cells play key roles in diabetic retinopathy and produce vascular endothelial growth factor (VEGF) that regulates retinal vascular leakage and proliferation. Melatonin is a potent antioxidant capable of protecting variety of retinal cells from oxidative damage. In addition to the pineal gland, the retina produces melatonin. In the current study, we investigated whether melatonin protects against hyperglycemia-induced oxidative injury to Müller cells and explored the potential underlying mechanisms. Our results show that both melatonin membrane receptors, MT1 and MT2, are expressed in cultured primary Müller cells and are upregulated by elevated glucose levels. Both basal and high glucose-induced VEGF production was attenuated by melatonin treatment in a dose-dependent manner. Furthermore, we found that melatonin is a potent activator of Akt in Müller cells. Our findings suggest that in addition to functioning as a direct free radical scavenger, melatonin can elicit cellular signaling pathways that are protective against retinal injury during diabetic retinopathy.

## Introduction

Diabetic retinopathy (DR) is a common and potentially devastating microvascular complication of diabetes; it is a leading cause of acquired blindness among people at occupational age [1], [2]. Nearly all patients with type I diabetes and over 60% of patients with type II diabetes will develop some degree of DR within 15 to 20 years after the initial diagnosis [2], [3]. Current therapeutic options including photocoagulation and vitrectomy are limited by their considerable side effects. Agents that antagonize VEGF are promising new treatment for proliferative diabetic retinopathy and macular edema; however, their long term safety and efficacy have yet been firmly established [4], [5]. Developing novel, mechanism-based therapeutic strategies is highly desirable for DR patients [1], [6].

Retinal angiogenesis is controlled by interactions between an array of growth factors and cytokines [1]. Among them, VEGF plays a crucial role in regulating vascular permeability and proliferation [1]. Müller cells, which are glial cells supporting the retinal neurons, have been recognized as a major source of VEGF production in the retina. Conditional knockout of Müller cell-derived VEGF reduced retinal angiogenesis and vascular leakage due to ischemia-reperfusion or hyperglycemia [7], [8]. The retina responds to hyperglycemic and hypoxic milieu by means of a number of biochemical changes. Dysregulated retinal VEGF production during DR is among the most devastating responses to oxidative stress [9], [10].

Melatonin, the main secretory product of the pineal gland [11], is a powerful endogenous antioxidant [12]–[14]. It easily crosses all membrane barriers due to its hydrophilic and lipophilic properties. As a result, melatonin has antioxidant properties at the level of cell membrane, mitochondria, and nucleus [15]–[17]. The major advantage of melatonin over classical antioxidants such as β-carotene, vitamin E and vitamin C, lies in its much stronger antioxidative effect and the lack of pro-oxidative actions [16]. Previous studies showed that hypoxic retina, which is a pathologic condition in DR, had lower melatonin content, and that dietary supplementation of melatonin inhibited VEGF production in the hypoxic retina [18]. In addition to the pineal gland, the retina is another site of melatonin synthesis [19] and its rate of synthesis decreased in streptozotocin-treated rats [20].

The goals of our current study were to investigate the protective effects of melatonin against hyperglycemia-induced VEGF production in primary rat Müller cells and to explore the underlying mechanisms. Our results show that in addition to its antioxidant functions, melatonin also activated key signaling pathways mediated by the phosphoinositide-3 kinase (PI3K) and nuclear factor erythroid 2-related factor 2 (Nrf2). The findings suggest that modulating these pathways with melatonin may have potential therapeutic implications for treating DR.

## Methods

### Isolation and Culture of Primary Rat Müller Cells

All animal procedures followed ARVO statement for the Use of Animals in Ophthalmic and Visual Research. Protocols were reviewed and approved by the Animal Ethics Committee of Fudan University (Shanghai, China). Sprague-Dawley rats (2–3 days old) were obtained from the Animal Center of Chinese Academy of Sciences (Shanghai, China). Retinas of 10−15 newborn rats were removed by microdissection under sterile conditions and pooled together. Cells were dissociated by 0.25% trypsin and collected by centrifugation at 500×g for 5 min. They were cultured in Dulbecco’s modified Eagle’s medium (DMEM) supplemented with 5.5 mM glucose, 20% fetal bovine serum (Hyclone, Logan, UT), streptomycin (100 µg/ml) and penicillin (100 µg/ml) in 25 cm^2^ culture flasks, maintained at 37°C in an incubator with a humidified atmosphere of 5% CO_2_. Culture medium was changed every 2 to 3 days. Cells at passages 2 to 4 were used for experiments [21]. Müller cells were confirmed by positive staining of their marker protein, glutamine synthetase [22] (data not shown).

### Exposure to High Glucose and Melatonin

Cultured Müller cells were divided into several groups, treated with either high glucose medium (containing 30 mM glucose) alone or combined with different concentrations of melatonin (10 nM to 0.1 mM) for various time. Melatonin was dissolved in dimethylsulfoxide (DMSO) and the stock solutions were added directly to the serum-free culture media. In the osmotic control group, cells were treated with medium containing 5.5 mM glucose and 24.5 mM mannitol. In some experiments, cells were pretreated with either a PI3K inhibitor, LY294002 (10 µM), or a melatonin receptor antagonist, luzindole (50 µM) (Sigma, St. Louis, MO).

### Immunocytochemistry

Cells were cultured on coverslips in 24-well plates. After different treatments, they were washed with phosphate-buffered saline (PBS) and fixed with 4% paraformaldehyde for 15 min. After methanol permeabilization, coverslips were blocked with 1% bovine serum albumin for 1 hr and then incubated overnight at 4°C in a humidified chamber with antibodies against melatonin receptors MT1 or MT2 (Santa Cruz Biotechnology, Santa Cruz, CA). Afterwards, the cells were washed three times with PBS/0.1% Tween 20 and then incubated with Alexa Fluor 488-conjugated secondary antibodies (1∶1000 dilution, Invitrogen, Grand Island, NY) for 1 hr at room temperature. After two further washes in PBS/Tween 20, the cells were further stained with 1 µg/ml of 4′,6-dianidino-2-phenylindole (DAPI) in PBS for 5 min to label the nuclei. The slides were mounted with glycerin and images were acquired with a Carl Zeiss LSM 510 confocal microscope.

### Measurement of Total Glutathione

Total cellular glutathione levels were measured using an assay kit (Beyotime Institute of Biotechnology, Haimen, China) which is based on DTNB (5,5′-dithiobis(2-nitrobenzoic acid)), the Ellman’s reagent, as the assay substrate. After treatment, harvested cells were deproteinated and centrifuged at 10,000×g for 10 min. Supernatants were added to a 96-well plate and assay was then performed according to the manufacturer’s instructions. DTNB reacts reduced glutathione (GSH) to generate 2-nitro-5-thiobenzoic acid (TNB), with peak absorbance at 412 nm. The other reaction product, the GSTNB mixed disulfide, is reduced back to GSH and TNB by glutathione reductase. The amount of total glutathione was calculated based on an external standard curve and normalized to the protein content in each sample.

### Enzyme-linked Immunoassay

After different treatments, Müller cell-conditioned media were collected and assayed for VEGF protein concentration with an ELISA kit (R&D Systems; Minneapolis, MN). For each experiment, a standard curve was constructed using 10–500 pg/ml of recombinant rodent VEGF. Data collected as absorbance was extrapolated to the standard curve to determine the concentration of VEGF per sample and were presented as the percentage relative to basal VEGF secretion.

### Real-time Quantitative PCR

Expression levels of the mRNA transcripts of MT1, MT2, catalytic and modulatory subunits of glutamate-cysteine ligase (GCLC, GCLM), heme oxygenase 1 (HO-1) and Nrf2 were measured by real-time RT-PCR. Total RNA was isolated (Trizol Reagent; Invitrogen), and cDNA was synthesized using M-MLV reverse transcriptase (Promega, Madison, WI) and random hexamer (Applied Biosystems, Foster City, CA). All quantitative PCR reactions were performed on an ABI 7300 system; using the SYBR Green-based detection method (Applied Biosystems). Primer sequences are listed in table 1. Average threshold cycle (Ct) values were used to determine the relative differences between control and treated groups, and were normalized to β-actin mRNA in each sample.

**Table 1:** Primer sequences for RT-PCR analyses.

Gene	Primer	Sequence	Product
MT1	For	5′-TCCTGTCTGTGTATCGCAACAAG-3′	338
	Rev	5′-GGGCATGATGGCTATGAGTG-3′	
MT2	For	5′-CCCCACAGCCTCTTCTTAGCACTTG-3′	169
	Rev	5′-CAGATGCACCAGTAGCGGTTGATG-3′	
HO-1	For	5′-ACAGGGTGACAGAAGAGGCTAAGAC-3′	243
	Rev	5′-GGAAACTGAGTGTGAGGACCCAT-3′	
Nrf2	For	5′-TATTTTCCATTCCCGAGTTACAGT-3′	288
	Rev	5′-CAGAGTAAAATTCATCACCGAAATC-3′	
GCLc	For	5′-AAGGTTGTCATCAATGTGCCAATA-3′	216
	Rev	5′-AGTGGCCAACTGGTCATAAAGGTAT-3′	
GCLm	For	5′-ACATGGCATGCTCAGTCCTT-3′	287
	Rev	5′-CTGTTTAGCAAATGCAGTCAAATCT-3′	
β-actin	For	5′-CTGAACCCTAAGGCCAACCGTGAAA-3′	274
	Rev	5′-TGAAGCTGTAGCCACGCTCGGTC-3′	

### Western Blot Analysis

Western blot analyses of MT1, MT2, phosphorylated Akt, Nrf2 and HO-1 were performed using specific antibodies. After treatment, cells were washed with PBS, harvested and lysed in RIPA buffer (50 mM Tris-Cl, pH 7.5, 150 mM NaCl, 1% NP-40, 0.5% DOC, 0.1% SDS). Equal amount of proteins were separated on 10% SDS polyacrylamide gels and transblotted onto polyvinylidene fluoride (PVDF) sheets (Immobilon TM-P, Millipore Corp., Bedford, MA, USA). All primary antibodies were obtained from Santa Cruz Biotechnology. Western signals were developed using HRP-conjugated secondary antibodies. Images were scanned from X-ray films and the band intensities were quantified with NIH Image J software.

### Statistical Analysis

All experiments were repeated at least three times. Data were analyzed with the SPSS 16 software (IBM). Means ± SD were calculated for each group. Significance of difference between two groups was evaluated using Student’s t-test. For multiple comparisons, one-way ANOVA followed by Tukey’s multiple comparison tests were used. Differences were considered statistically significant when p-value was less than 0.05.

## Results

### High Glucose-induced Upregulation of MT1/MT2 and HO-1 Expression in Müller Cells

**Figure 1:**
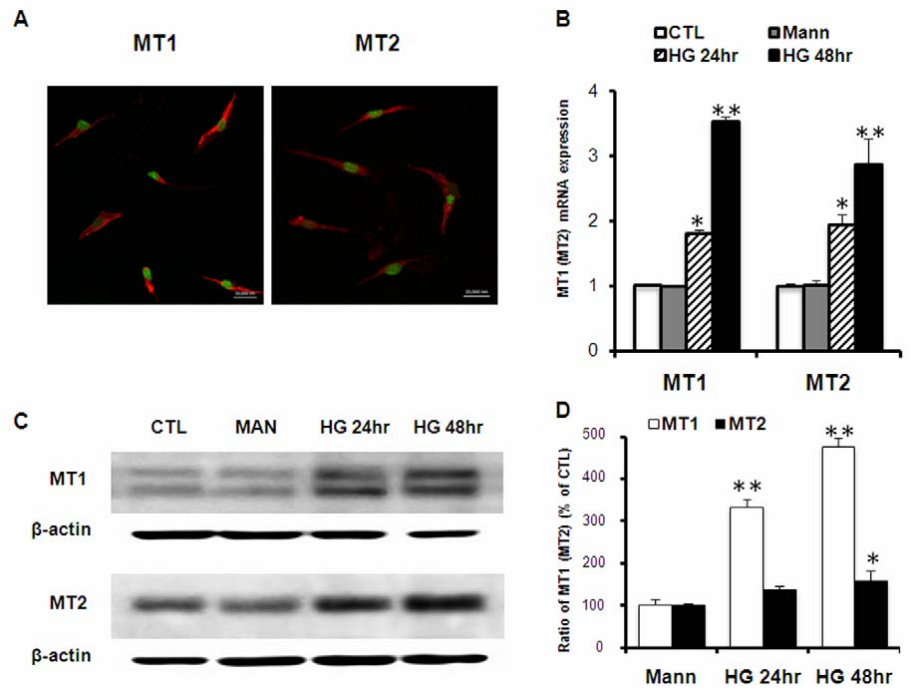
High glucose-induced upregulation of melatonin receptors in Müller cells. (A) Immunostaining of MT1 and MT2 in cultured primary rat Müller cells. Nuclei were counterstained with SYTO green, Scale bar = 20 µm. (B) High glucose-induced mRNA expression of both melatonin receptors. Cells were treated with 30 mM glucose for 24 and 48 hr. MT1/MT2 expression was measured by Real-time RT-PCR and normalized to β-actin mRNA. CTL: untreated control; Mann: 24.5 mM mannitol as osmolarity control. (C) Western blot of MT1/MT2 proteins in Müller cells treated with high glucose. (D) Quantification data from the Western blots after normalizing to β-actin. Data presented are the average from 3 to 5 separate experiments (mean ± SD). *P<0.05, **P<0.01, as determined by either one-way ANOVA (B) or Student’s t-test (D).

Previous studies have shown that melatonin membrane receptors were distributed in the retina including photoreceptor, inner nuclear layer (INL) and ganglion cell layer (GCL) [23]. The bodies of Müller cells sit in the inner nuclear layer and they appear to respond to melatonin treatment *in vivo* [18]. As shown in Figure 1, cultured primary rat Müller cells expressed both MT1 and MT2 receptors (Figure 1A), and their mRNA and protein levels were upregulated by high glucose (Figure 1B and 1D) in a time dependent manner. After 48 hr of high glucose treatment, the mRNA level of MT1 and MT2 increased 3.53±0.08 and 2.87±0.14 folds, respectively (Figure 1B) (P<0.05, one way-ANOVA and Tukey’s post-hoc test). The protein level of MT1 rose significantly at 24 hr; however, level of the MT2 protein only elevated slightly at 48 hr after exposure to high glucose (Figure 1C and 1D). Such difference is consistent with the previously reported *in vivo* study [24] and indicates a potentially more direct role of MT1 in regulating the cellular responses to high glucose condition. The double bands of MT1 on Western blots were likely due to the post-translational modification of the receptor protein [25]–[26]. Meanwhile, cells treated with mannitol as osmolality control did not show changes in either mRNA or protein levels of melatonin receptors (Figure 1B–D). Thus, cultured primary Müller cells express both MT1 and MT2 receptors which are upregulated in hyperglycemic conditions.

### Melatonin-mediated Protective Effects Against Hyperglycemia

**Figure 2:**
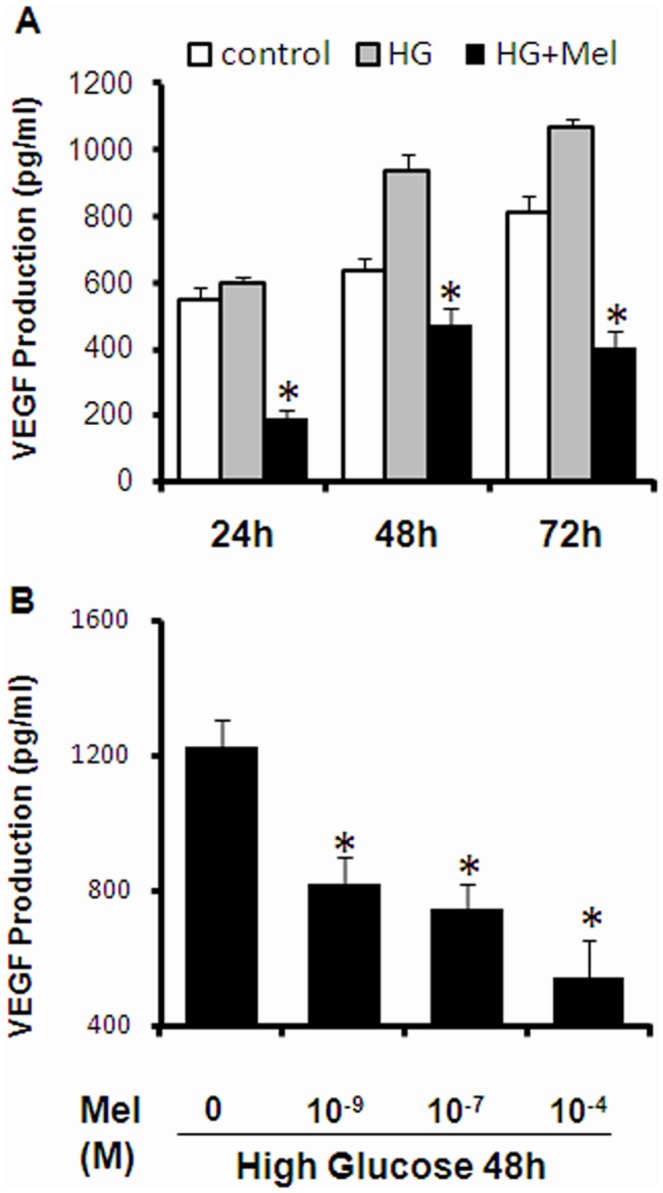
Melatonin-mediated inhibition of VEGF production from Müller cells. (A) Cells were treated with either 30 mM glucose (HG) or glucose and 100 µM melatonin (HG+Mel) for indicated time. The amount of VEGF secreted in the conditioned media was measured by ELISA. (B) Dose-dependent inhibition of VEGF production by melatonin. VEGF production was measured at 48 hr after high glucose stimulation in the presence of different concentrations of melatonin. Data presented are the average of 2 separate experiments. *P<0.05 one-way ANOVA and Tukey’s post-hoc test.

Retinal angiogenesis is a major complication of diabetes and the condition is controlled by VEGF which is presumably produced from Müller cells. We evaluated whether melatonin can suppress excessive secretion of VEGF from Müller cells in response to high glucose treatment. As expected, hyperglycemia stimulated VEGF production in Müller cells in a time-dependent manner. Co-treatment with melatonin significantly reduced the amount of released VEGF by more than 50% at all time points (Figure 2A). Furthermore, the effects of melatonin were concentration-dependent, as the greatest VEGF inhibition was achieved by 100 µM of melatonin (Figure 2B). Thus, melatonin attenuated the VEGF overproduction induced by hyperglycemia in time- and concentration-dependent manner.

**Figure 3:**
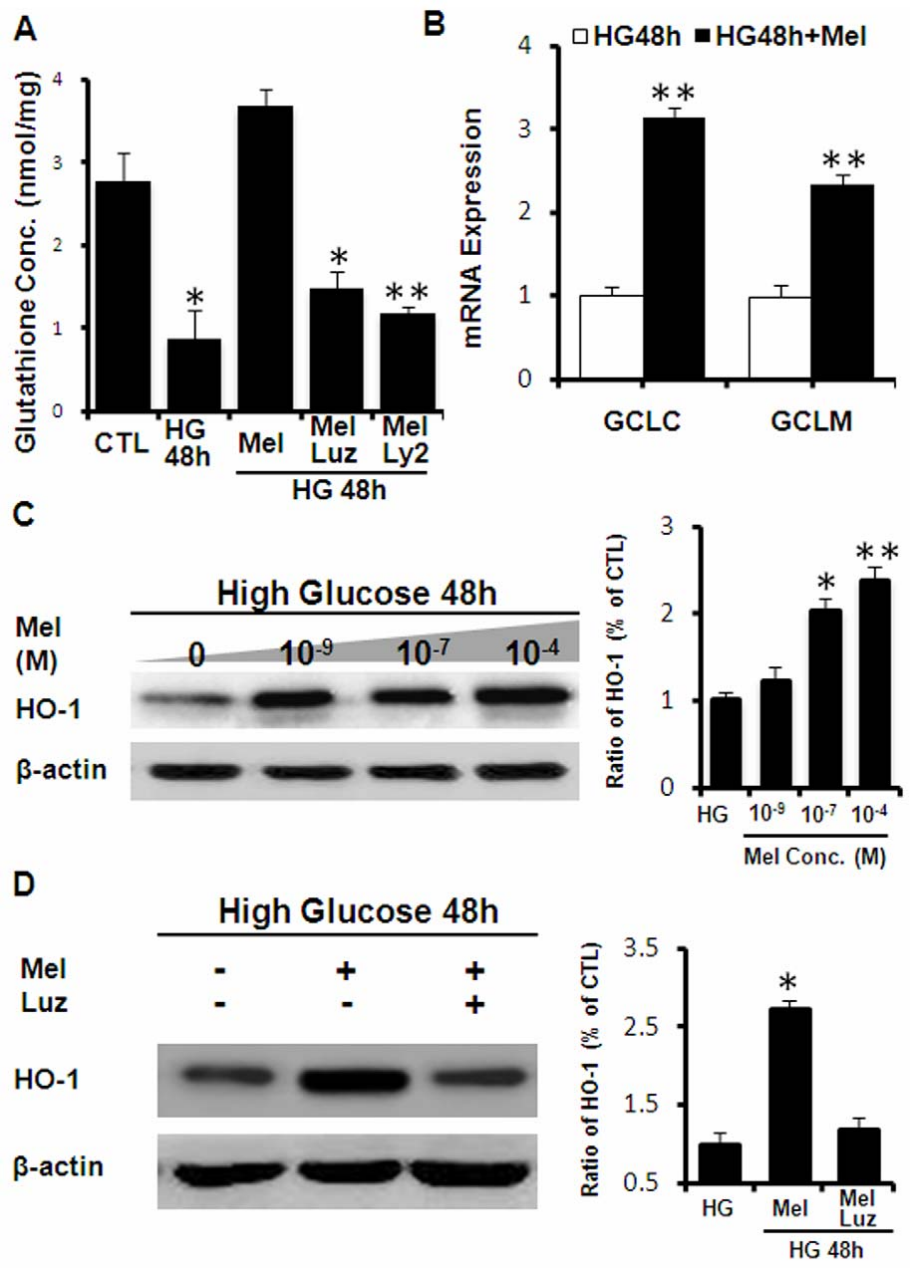
Protection against high glucose-induced oxidative stress by melatonin. (A) Measurement of cellular glutathione contents in Müller cells exposed to high glucose. Cells were treated with 30 mM glucose (HG) alone or in together with 0.1 mM melatonin (Mel), 50 µM luzindole (Luz) or 50 µM LY294002 (Ly2). Glutathione was measured by the DTNB method. (B) Upregulation of GCLc and GCLm measured by real-time RT-PCR and normalized to β-actin mRNA. (C) Western blot analyses of HO-1 expression after co-treatment with high glucose and indicated concentrations of melatonin (Mel). (D) Effects of luzindole on melatonin-induced upregulation of HO-1. Data presented are average from 3 to 5 experiments (*P<0.05; **P<0.01).

Oxidative stress is a common biochemical mechanism underlying the various pathological effects of hyperglycemia including VEGF overproduction. We measured cellular glutathione content as a marker of oxidative stress in Müller cells treated with high glucose. The results (Figure 3A) showed that control cells had 2.77±0.35 nmol/mg protein of total glutathione. High glucose treatment for 48 hours decreased glutathione content by 60% to 0.86±0.31 nmol/mg protein. In contrast, co-treatment with melatonin completely prevented the glutathione depletion induced by hyperglycemia. Furthermore, the effects of melatonin on glutathione level in Müller cells were blocked by luzindole, which was a nonselective melatonin receptor antagonist.

High glucose-induced oxidative stress in Müller cells is resulted from an imbalance between the production of reactive intermediates and the protection/clearance by the cellular antioxidant systems. We measured changes in the expression of a number of cytoprotective genes encoding antioxidant and detoxification enzymes in response to glucose and melatonin treatment. The results showed that melatonin potentiated high glucose-induced upregulation of both the GCLC and GCLM, the rate limiting enzyme of glutathione synthesis (Figure 3B). Such effects were also inhibited by luzindole (data not shown), indicating receptor-mediated signaling. In addition, melatonin also increased HO-1, a major antioxidant protein involved in biliverdin and bilirubin synthesis (Figure 3C and 3D). Western blot data revealed that the level of HO-1 protein was significantly elevated by co-treatment of high glucose and melatonin in a dose-dependent manner (Figure 3C). Supplementation of 100 µM melatonin under high glucose condition for 48 hours increased HO-1 protein by 2.39±0.16 folds as compared to the group treated with high glucose alone. Again, such an effect could be completely blocked by luzindole (Figure 3D). Collectively, the data suggest that melatonin can exert protective effects in Müller cells against high glucose-induced oxidative stress and VEGF overproduction.

### Signaling Pathways Modulated by Melatonin in Müller Cells

**Figure 4:**
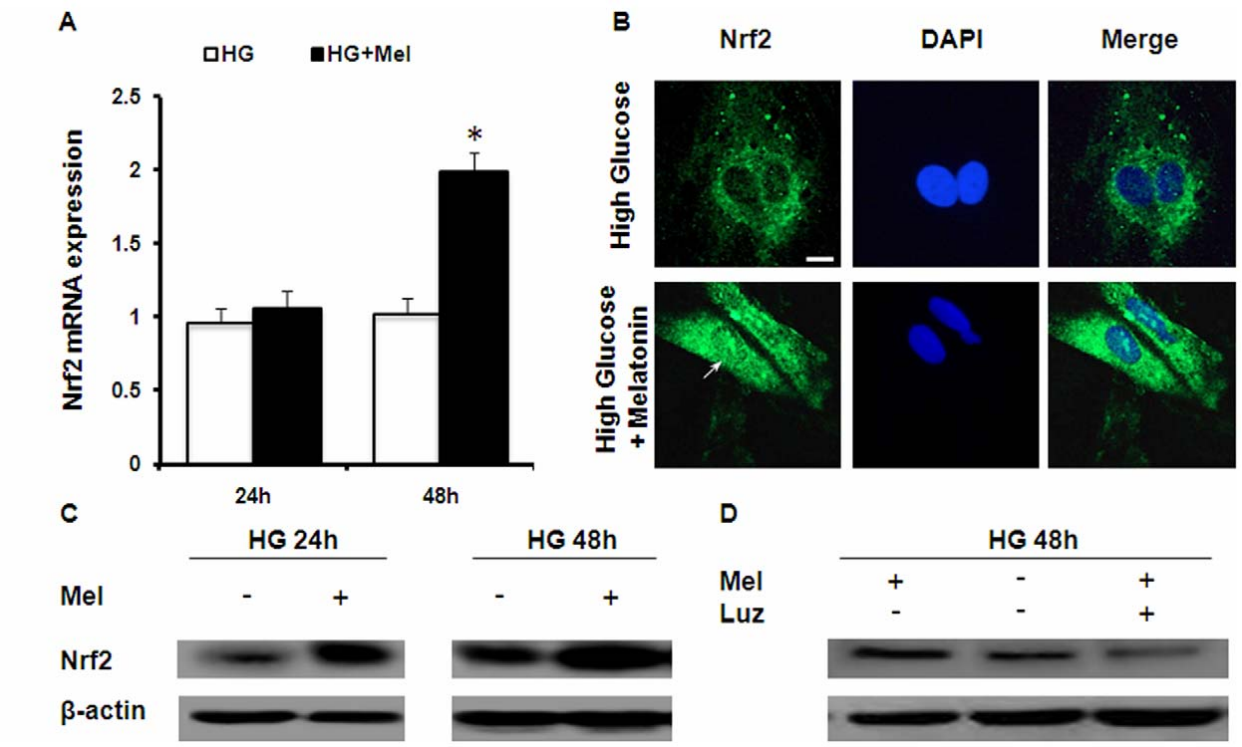
Effects of melatonin on Nrf2 expression and localization in Müller cells. (A) The mRNA level of Nrf2 was measured by real-time RT-PCR after 24 and 48 hr exposure to high glucose with or without melatonin. (B) Immunostaining of Nrf2 localization after high glucose and melatonin treatment. The nuclei were counterstained with DAPI. Scale bar = 5 µm. (C) Western blot analyses of Nrf2 protein after 24 and 48 hr treatment. (D) Effects of luzindole on Nrf2. Data presented are representative of three independent experiments. (*P<0.05).

Nrf2 is a master regulator of cellular antioxidant response and is a transcriptional factor that controls the expression of phase 2 genes like GCL and HO-1 [27], [28]. Real Time RT-PCR data showed that expression level of Nrf2 was elevated in hyperglycemic Müller cells when treated with melatonin (Figure 4A). The increase was observed at protein level as shown by the Western blot analyses (Figure 4C). In addition, we used immunocytochemistry to measure the nuclear translocation of Nrf2 after melatonin treatment. As shown in Figure 4B, Müller cells challenged with high glucose for 48 hours did not elicit nuclear staining of Nrf2. However, co-treatment with melatonin induced redistribution of Nrf2 in the nucleus which was colocalized with DAPI fluorescence. Luzindole inhibited the effects of melatonin on Nrf2 (Figure 4D). Together, these data suggest that in addition to being a potent scavenger of free radicals, melatonin also activates the endogenous antioxidant system in Müller cells via Nrf2-dependent mechanisms.

**Figure 5:**
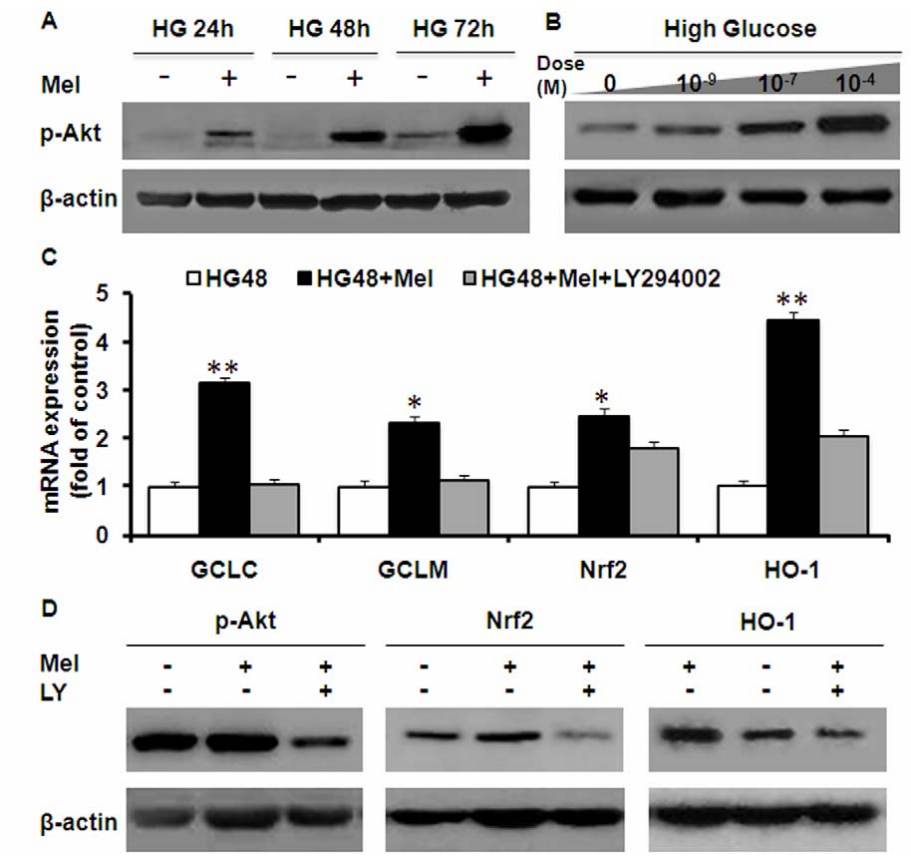
Effects of melatonin on Akt activation in Müller cells. (A) Cells were treated with 30 mM glucose either with or without 0.1 mM melatonin for indicated time. Akt phosphorylation was measured by Western blot analyses. (B) Dose-dependence of melatonin on Akt phosphorylation. (C) Effects of high glucose, melatonin and PI3K inhibitor LY294002 on antioxidant gene expression, as measured by real-time RT-PCR. (D) Effects of LY294002 on melatonin-mediated protective responses in Müller cells. Cells were treated with 30 mM glucose, melatonin or LY294002 for 48 hr. Akt phosphorylation, and the protein levels of Nrf2 and HO-1 were measured by Western blot analyses. (*P<0.05; **P<0.01).

The serine/threonine kinase Akt is a key regulator of diverse cellular processes including insulin signaling and glucose metabolism. Melatonin has been reported to either activate [29]–[30] or inhibit Akt [31]–[32] depending on the cell type and dosage. In primary Müller cells, we observed a time and dose-dependent increase of Akt phosphorylation in melatonin-treated cells compared to its high glucose counterparts (Figure 5A and B). Furthermore, LY294002, a specific inhibitor of PI3K, significantly decreased Akt phosphorylation, GCL, Nrf2 and HO-1 expressions at 48 hr after exposure to melatonin treatment (Figure 5C and D). LY294002 also inhibited the effects of melatonin on cellular glutathione content (Figure 3A). Thus, melatonin can promote major cell survival and antioxidant pathways that are associated with hyperglycemia-induced biochemical changes in Müller cells.

## Discussion

In this study, we demonstrated the *in vitro* protective effect of melatonin in cultured primary Müller cells under hyperglycemic conditions. We showed that melatonin attenuated high glucose induced VEGF overproduction (Figure 2) and glutathione depletion (Figure 3A). Oxidative stress is a signal stimulating VEGF production. We showed that melatonin exhibited indirect antioxidant effects by enhancing the expression of various antioxidant genes, including GCLC, GCLM, HO-1 and Nrf2 (Figure 3 and Figure 4), which contributed to maintaining the cellular redox status and preventing against oxidative injury. Finally, we showed that melatonin activated PI3K pathway, which led to increased Akt phosphorylation in Müller cells (Figure 5).

Müller cells, the principal glial cells of the retina, become activated promptly upon virtually all pathogenic stimuli [33]. Gliosis is considered as a cellular response to protect the neuronal tissue from further damage and often results in increased release of neurotrophic factors and antioxidants [34]–[36]. Diabetes-induced Müller cell gliosis is an early response to diabetic retinal milieu caused by ischemia-hypoxia, vascular leakage and oxidative stress. Müller cell is a major source of retinal VEGF production. Although low concentration of VEGF is part of a pro-survival signaling in the hypoxic tissue with actions that include vasodilatation, endothelial cell survival, neuroprotection, neurogenesis [37], high concentration VEGF contributes retinal neovascularization and vascular leakage, which causes macular edema and proliferative diabetic retinopathy, respectively. Research by Bai et al [38] suggested that the retinal Müller cell-derived VEGF is a major contributor to ischemia induced retinal vascular leakage, and pre-retinal and intra-retinal neovascularization. Results from our current study suggest that melatonin effectively inhibited high glucose-induced VEGF production from Müller cells (Figure 2). Such finding suggests a novel therapeutic effect of melatonin in DR.

Melatonin membrane receptors, MT1 and MT2, are widely distributed in various mammalian tissues [39]. MT1 receptors mediate vasoconstriction, cell proliferation, reproduction and metabolism, while MT2 receptors regulate dopamine release, vasodilation, leukocyte migration and immune responses [40]. Both melatonin receptors have been localized to the inner and outer nuclear layers, and ganglion cell layer by in situ hybridization and immunostaining [41]–[42]. However, given the diversity of cell types that exit in these layers, the cell type-specific distribution of MT1/MT2 receptors in either control or diabetic animals remains unclear and needs to be further investigated by future studies. Also the effects of melatonin can be either receptor-dependent [43] or independent [40]. In the present study we report the expression of both MT1 and MT2 on primary rat Müller cells, as detected by RT-PCR, Western blot and immunocytochemistry (Figure 1). Such finding is in consistent with previous report of Müller cells as a major target for melatonin in hypoxic retina [18]. Furthermore, the melatonin receptor antagonist luzindole effectively blocked most of the protective effects, indicating the antioxidative and anti-angiogenic effects of melatonin in Müller cells are largely receptor-dependent.

The PI3K/Akt pathway is a major regulator of cell survival pathway [44] and also controls Nrf2-mediated antioxidant function [45]–[46]. We showed that melatonin effectively activated Akt in Müller cells in a time-dependent manner (Figure 5A). Similar findings have been reported in other cell types [29]. The PI3K inhibitor, LY294002, eliminated all antioxidant effects, such as prevention of GSH depletion and antioxidant gene regulation (Figure 3 and Figure 5). Mechanisms by which melatonin activates Akt will have to be determined in future studies, but will likely mediated by its receptors MT1/MT2.

In summary, data from our study showed that melatonin reduced the induction of VEGF secretion and augments cellular antioxidant defense capacity through HO-1 and GSH induction via the PI3K/Akt-Nrf2 signaling pathway. Melatonin is considered as an ideal antioxidant drug for its high efficiency, safety, easy of synthesis in a pharmacologically pure form and its affordability [47]. In addition to its protective effects in Müller cells during DR, melatonin can prevent oxidative injury and cell death in retinal pigment epithelial (RPE) cells which are implicated in age-related macular degeneration (AMD) [48]. Thus, melatonin is a potent antioxidant compound with potential for therapeutic applications in those common retinal diseases.
